# An integrated primary care service to reduce cardiovascular disease risk in people with severe mental illness: Primrose-A - thematic analysis of its acceptability, feasibility, and implementation

**DOI:** 10.1186/s12913-024-10628-6

**Published:** 2024-02-28

**Authors:** Philippa Shaw, Annabel Mifsud, David Osborn, Nitisha Nahata, Cerdic Hall, Ian Prenelle, Danielle Lamb

**Affiliations:** 1 Division of Psychiatry, University College London, 6th Floor, Maple House, 149 Tottenham Court Road, W1T 7NF London, United Kingdom; 2 Department of Applied Health Research, UCL, 1-19 Torrington Place, WC1E 7H London, United Kingdom; 3 Camden and Islington NHS Trust, 4 St Pancras Way, NW1 0PE London, United Kingdom; 4 Camden Health Partners LTD, St Pancras Hospital, 4 Saint Pancras Way, South Wing, NW1 0PE London, United Kingdom

**Keywords:** Severe mental illness, Psychosis, Behaviour, Primary health care, Program evaluation, Qualitative research, Cardiovascular disease prevention

## Abstract

**Background:**

Cardiovascular disease among patients with severe mental illness in England is a major preventable contributor to premature mortality. To address this, a nurse and peer-coach delivered service (Primrose-A) was implemented in three London general practices from 2019 (implementation continued during COVID-19). This study aimed to conduct interviews with patient and staff to determine the acceptability of, and experiences with, Primrose-A.

**Methods:**

Semi-structured audio-recorded interviews with eight patients who had received Primrose-A, and 3 nurses, 1 GP, and 1 peer-coach who had delivered Primrose-A in three London-based GP surgeries were conducted. Reflexive thematic analysis was used to identify themes from the transcribed interviews.

**Findings:**

Overall, Primrose-A was viewed positively by patients and staff, with participants describing success in improving patients’ mental health, isolation, motivation, and physical health. Therapeutic relationships between staff and patients, and long regular appointments were important facilitators of patient engagement and acceptance of the intervention. Several barriers to the implementation of Primrose-A were identified, including training, administrative and communication issues, burden of time and resources, and COVID-19.

**Conclusions:**

Intervention acceptability could be enhanced by providing longer-term continuity of care paired with more peer-coaching sessions to build positive relationships and facilitate sustained health behaviour change. Future implementation of Primrose-A or similar interventions should consider: (1) training sufficiency (covering physical and mental health, including addiction), (2) adequate staffing to deliver the intervention, (3) facilitation of clear communication pathways between staff, and (4) supporting administrative processes.

**Supplementary Information:**

The online version contains supplementary material available at 10.1186/s12913-024-10628-6.

## Background

Patients with severe mental illness (SMI; schizophrenia, bipolar disorder, major depressive disorder, and related spectrum disorders), have been predicted to die 17.5 years earlier than the general population [1]. Whilst this can be partially attributed to SMI-related deaths including suicide, physical comorbidities are estimated to cause approximately 60% of this mortality gap [1, 2].

Patients with SMI generally develop physical health problems earlier and more frequently [3, 4], with cardiovascular disease (CVD; physical health problems of the heart or blood vessels), obesity, type 2 diabetes, respiratory diseases, cancer, and infectious diseases being prevalent among patients with SMI [2, 5]. Patients with SMI have a higher risk of developing (78%) and dying from CVD (85%) compared to the general population [2, 5].

Concerningly, between 2000 and 2014 the mortality gap widened for patients with bipolar disorder and schizophrenia in the UK [6]. Potentially contributing to this is suboptimal diagnosis and treatment of physical diseases for patients with SMI [7], which has been linked to healthcare provider stigma around mental illness, systemic issues, limited appointment time, a lack of training, and fragmentation between primary and secondary care [5, 8]^,^ [9]^,^ [10, 11].

The NHS Long Term Plan [12] pledged a growing share of the NHS budget towards mental health to help form ‘Integrated Care Systems’ for delivering more coordinated care. A foundational idea across this Plan is to empower patients to look after their own health, such as with targeted weight management support and investment in social prescribing. Research suggests that if modifiable CVD risk factors (such as being overweight or smoking) are appropriately addressed, CVD can be largely prevented [1, 5, 13, 14].

The NHS has committed to providing physical health screening for patients with SMI in primary care [15], but evidence indicates a need for physical disease prevention for patients with SMI too. Therefore, there is need for targeted, integrated interventions which can address CVD risk once recognised among patients with SMI in physical health screenings to improve quality of life [10, 11, 16].

### Primrose

The Primrose intervention was a 6-month nurse-led primary care service developed to manage CVD risk in people with SMI. Patients received approximately eight 30-minute appointments which incorporated behavioural change strategies to reduce CVD risk factors; patients were supported to set goals (such as improving diet), track progress, form habits, and overcome setbacks. Whilst Primrose was focused on the reduction of CVD risk, the service emphasised integrated care to also support their mental health.

Primrose was evaluated in a cluster randomised trial across GP surgeries in England [17], with findings suggesting that Primrose could modify CVD risk in patients with SMI. Compared to screening and feedback on CVD risk (standard care), Primrose was estimated to be able to save £895 in healthcare costs per patient over 12 months through reducing psychiatric inpatient costs, adverse events, and hospital admissions.

However, the trial did not show a difference in total cholesterol between intervention and standard care. This may be due to limitations in trial design and duration [17], better than typical general care in standard care, heterogeneous intervention delivery, or suboptimal emphasis on statins [18]. Further Primrose research has suggested higher perceived social support may facilitate statin adherence and appointment attendance [19]. Therefore, potential enhancements to improve physical outcomes would be to integrate an emphasis on statins and expand the potential for social support through the intervention.

### Primrose-A

Building on the Primrose trial findings, the intervention was adapted (Primrose-A). The adaption aimed to further augment the holistic, integrated approach to mental and physical health improvement and prevention. For example, patients were encouraged to set psychosocial goals in addition to their physical health goals, which aimed to achieve a less dualistic approach to the interactions between mental and physical health faced by people with SMI.

Furthermore, Primrose-A had an increased focus on the prescription of statins and a supplementary offer of up to four 60-minute appointments with a peer-coach who had lived experience of SMI. These appointments included discussing goals, personal experiences, and plans for activities. Peer coaches bring a unique skillset and approach to their support which can have a positive impact [20]. In this model, mental health nurses and Voluntary and Community Sector staff are embedded within primary care, thereby strengthening links between primary and secondary care providers and statutory and non-statutory services, making this a ‘boundary-spanning’ collaborative innovation [21].

Primrose-A was implemented in GP surgeries in December 2019. The COVID-19 pandemic led to a further adaption, with the standard template used by healthcare professionals to record Primrose-A consultations developed to include a prompt to ask patients about how they were coping during the pandemic, and sessions were provided virtually. Whilst there are potential advantages to virtual delivery, research suggests barriers towards virtual care such as low enthusiasm, privacy concerns [22], and disparities in engagement [23] remain.

This research study aimed to determine the acceptability of, and experiences with Primrose-A, and explore factors that influenced the implementation (including COVID-19), feasibility, and continuation of Primrose-A.

## Methods

### Study design and setting

Interviews were carried out with staff and patients from three London-based GP surgeries which implemented Primrose-A. This study design was reviewed by the NIHR North Thames Research Advisory Panel (members provided patient, public, or carer perspectives), with feedback incorporated into the patient information leaflets, interview guides, and consent forms.

### Participants

Recruitment took place between February and May 2021. Eligible participants were 18 or older, able to provide informed consent and engage in an interview, and had delivered (staff) or received (patients) Primrose-A.

Potential patient participants were contacted by Primrose-A staff and informed about the study. If willing to participate, patients provided permission to be contacted by the researchers. The researchers then provided further information about the study and organised a telephone interview for at least 24-hours after initial contact for eligible patients.

All staff involved in managing and delivering Primrose-A at the three GP surgeries were invited to participate in this study. Invites were issued directly by the research team to staff via emails and in meetings. Staff involved included a total of three GPs, three nurses, and eight peer coaches. GPs and nurses each worked in only one of the GP surgeries, while peer coaches worked across the three GP surgeries. Information about the study was provided to all involved staff, and interested members of staff were invited to contact researcher directly, at which point telephone or video calls were arranged. There was no relationship between researchers and participants before the study.

A purposive sample of 8 patients and 5 staff were recruited (due to the small sample, a detailed breakdown of demographics has not been provided). Patient ages ranged from 40 to 49 to 70–79, most self-identified as female [5], and self-identified as White [5], Mixed White [2], or African/African British [1]. Staff participants included 1 peer-coach, 3 nurses, and 1 GP. Staff ages ranged from 40 to 49 to 60–69, most self-identified as female [4] and identified as White [4] or Mixed white [1].

### Data collection

On the day of the interview, written consent and demographic characteristics were recorded using Microsoft Forms on behalf of each participant, and verbal consent was recorded using a dictaphone. Audio recorded semi-structured interviews (see additional file 1 for topic guides), conducted by AM and DL (supervisor) ranged from 13 to 43 min (average of 29 min). Patients were offered a £20 gift voucher as a thank-you.

### Data analysis

An inductive reflexive approach was used for analysis, informed by Braun and Clarke’s six-phase process of thematic analysis [24, 25]. This approach was consistent [26–28] with the authors’ acknowledgement of the influence of preconceived theories, experience, subjectivity in research, their epistemological orientation of critical realism, and a preference to actively embrace questioning and reflecting through analysis.

For the analysis, AM and DL familiarised themselves with the transcripts through re-reading and making notes; transcripts were coded systematically by AM using NVivo (Version 12); provisional themes generated from the codes with data extracts were discussed with DL and the wider team (including staff delivering the intervention, some of whom were interview participants) at several meetings; these themes were further developed and explored using a reflexive process and reviewed regularly with DL to ensure alignment with the study aims and fidelity to the primary data; themes were named; and finally, these themes brought together into a narrative [24, 28, 29].

### Reflexivity

The researchers engaged with reflexivity [24, 25] which entailed spending time being actively self-aware of previous experiences, positionality, and understanding of SMI and CVD, then exploring the potential influence on the findings. AM previously had little experience of working with people with SMI, so discussed her assumptions about SMI with DL, who has over a decade of experience of working in this area. Both reflected on their personal experiences of knowing people with common mental disorders, and how this might influence the research. How and why themes were identified were questioned during the analysis, and the thematic framework was discussed between AM and DL to minimise confirmation bias. Moreover, the researchers spent time reflecting about both being white women, and how that might be influencing interpretation.

## Findings

This section is structured to address the main objectives: (i) grouping themes under implementation and feasibility; (ii) and then acceptability and experience. A theme map is available in Additional file 2.

### Implementation and feasibility

Participants discussed the process of implementing, delivering, and taking part in Primrose-A. Several facilitators and barriers were discussed, relating to knowledge and beliefs, and accessibility.

### Knowledge and beliefs

In general, the training to support staff delivering Primrose-A was described as useful. However, some staff placed more value on learning through practice:

*“The training was fine, it gives you a background… but you learn so much more when you actually see the patients.”* (Staff 4, nurse).

Several staff reported limitations in the depth of content provided, especially when staff had varying existing experiences, knowledge, and approaches to work: *“I think we both work very differently in how we would approach the same problem”* (Staff 3, nurse). The need for specialised training in substance abuse was mentioned by one patient, *“You need to have someone who knows a bit about that subject”* (Patient 7, female).

Additionally, several patients viewed Primrose-A as primarily a mental health intervention, *“Just to touch base and see whether my mental health condition was having a greater or lesser effect”* (Patient 5, male), misunderstanding that Primrose-A was a holistic approach which supported patients’ mental health, wellbeing, and physical health, with staff reporting that they had to reinforce this as the wider focus of Primrose-A to patients.

### Accessibility

There was concern that not all eligible patients could receive Primrose-A, which was generally viewed as a beneficial intervention. Staff discussed the inclusion criteria prevented patients with different diagnoses accessing the services:

*“There might be people who’d really benefit from the Primrose intervention, but they might not fall under the heading of an SMI.”* (Staff 3, nurse).

Moreover, a key barrier to accessibility described by staff was COVID-19. Most staff preferred face-to-face appointments for the opportunity to track physical health progress and the challenges of providing Primrose-A by telephone for patients who did not speak English as their first language. One nurse emphasised language services were difficult to access and there was low opportunity for the “*Continuity of interpreter as well as the nurse”* (Staff 2, nurse).

In contrast, patients’ views on telephone appointments were mixed, with some disliking the impersonal feel of phone calls, *“You don’t see somebody’s expression on their face”* (Patient 3, female), or feeling that even video calls were not sufficient, *“It’s still really not quite the same as seeing that person”* (Patient 8, male). But most patients appreciated the telephone delivery, particularly if their SMI symptoms could impact travel to an appointment: *“I got very paranoid… Having it done by phone, especially at the time, was very useful”* (Patient 5, male).

There were several structural barriers to the implementation including the time and resource investment needed. This was apparent in the planning, recruitment, and provision stages:

*“I think the on-going provision of multiple appointments is hard within the practice framework.”* (Staff 2, nurse).

Additionally, staff felt the set-up, provision of materials, reimbursement, and organisation was problematic:

*“There’s very little spare time shall we say in the practice, for actually doing any admin… It would have been nice to perhaps, if those things had been clearly done and were available when we first did it.”* (Staff 2, nurse).

It was suggested that there needed to be better communication between those managing and delivering the intervention. Moreover, a lack of communication between staff was reported, which impacted the provision of holistic care:

*“If you’re lucky, you get a name of a peer support worker, [but] you’re unlikely to get any way of contacting them.”* (Staff 1, GP).

However, one nurse did highlight that in practice, Primrose-A *“Decreased the workload for GPs*” (Staff 4, nurse) by providing patients with frequent, targeted appointments to address their mental and physical health, rather than relying on GP care.

### Acceptability and experience

Despite the barriers to implementation, Primrose-A was viewed as valuable in supporting patients’ mental, physical, and social needs, particularly during the pandemic. This theme highlights perceptions that patients received the right support at the right time, facilitated through positive therapeutic relationships, which led to good overall engagement.

### The right support at the right time

Staff and patients recognised that patients may experience increased SMI symptoms during the COVID-19 pandemic, *“People’s mental health became quite a potential issue”* (Staff 1, GP). Patients described Primrose-A as support offered *“At the most opportune, perfect time”* (Patient 2, male), with most participants associating Primrose-A with an opportunity for needed additional support: *“She was able to keep me focused”* (Patient 1, female).

Importantly, there was the potential to address patients’ social needs, with isolation commonly mentioned. The impact of COVID-19 on isolation was compounded by some patients’ reluctance to include their family or friends in their support system, *“They’ve got their own lives… I don’t put any of [my] problems on them”* (Patient 8, male), or to consult GPs because of time constraints: *“I never like spending that long because I know how much of [a] backlog she’s got”* (Patient 7, female). Therefore, patients commonly valued Primrose-A staff, *“It’s just nice to have somebody”* (Patient 7, female). This perspective was recognised by most staff who understood their role as a point of contact for patients when they may be feeling isolated: *“The only people they ever talked to was me really”* (Staff 4, nurse).

This positive impact was boosted through connection with peer-coaches, with the peer-coach noting the supportive relatability and informal nature of their sessions: *“If that client can relate to someone it definitely makes a difference”* (Staff 5, peer-coach). A strong, trusting therapeutic relationship between patients and staff, where the patients’ perspectives were respected was mentioned by most to help with acceptance:

*“It’s about feeling listened to, and if they’re listened to and you’ve took that onboard, then let’s do that together it kind of seems an easier thing or less scary perhaps for them.”* (Staff 2, nurse).

For some participants it was particularly helpful that they were matched with peer coaches who had experiences similar challenges to those they were going through:

*“He understood exactly my problem, so, he… yeah, just loads of recommendations. Some of them weren’t quite right for me, but some of them really were”* (Patient 4, female).

### Engagement

By creating an intervention that fostered positive therapeutic relationships, and addressed patients’ additional needs, Primrose-A was perceived positively, which increased engagement. For example, patients described how the nurses’ non-judgemental attitude helped them to persevere in pursuing their goals:

*“She was quite perceptive and there was no judgement, you know, it was just, okay…and tried to see that there is a solution that might work and then try that the next week.”* (Patient 4, female).

All participants described their experience of the intervention favourably and most patients reflected that they would recommend Primrose-A. Primrose-A was associated with the benefits of long-term continuous care facilitating honest discussions, progress tracking, and encouraging healthier behavioural changes. Staff additionally reported positive patient feedback, that their knowledge of SMI had improved, and the rewarding nature of the intervention:

*“To actually see people on that journey and achieve things… It was as rewarding for me as it was to the client group.” (Staff 2, nurse)*.

Staff viewed Primrose-A as a way to deliver patient-centred, tailored care which helped patients engage with goal setting, a core component of Primrose-A. Flexibility was highlighted by both staff and patients as a key facilitator of patient engagement:

*“It allows a fair amount of flexibility, people, patients could choose their goals, rather than being sort of put into the same ones.”* (Staff 1, GP).

Common patient goals were to generally *“Become a healthier person”* (Patient 7, female), go outside, and exercise:

*“A lady I had on the study in particular did absolutely fantastic… She joined a yoga class online, she went walking in the heat, she’d never done any of this before.”* (Staff 4, nurse).

Staff therefore frequently discussed associating Primrose-A with physical health benefits, but also mental health and general wellbeing.

Lack of patient engagement was infrequently discussed, but when disengagement occurred, staff reflected on patients’ personal barriers such as navigating challenging mental health symptoms or patient personalities: *“I think that was more about the type of people they were”* (Staff 4, nurse).

A common theme among staff and patients was the desire for more Primrose-A appointments to encourage further progress and long-lasting changes, with patients appreciating the support, and staff wanting to spend time *“Getting to know the client”* (Staff 5, peer-coach). Patients expressed a need for follow-up appointments and more sessions with the peer-coaches, highlighting the meaningful positive impact of long-term care. One patient also suggested a need for more clarity at the end of Primrose-A on the next steps: *“Did she feed back to my GP at all, I don’t know*” (Patient 7, female).

## Discussion

Integrated mental and physical healthcare has been frequently suggested as an important strategy to improve the health of patients with SMI and reduce the widening mortality gap, however few of these services exist [30]. Primrose and Primrose-A attempt to address this need, focusing on targeting CVD risk factors in patients with SMI. To improve the physical health outcomes of Primrose, Primrose-A had a larger focus on statin prescription and peer support, whilst also responding to the contextual factors of COVID-19.

Participants perceived Primrose-A as an acceptable and feasible primary care intervention and discussed that, despite the barriers caused by COVID-19, the behavioural change components of Primrose-A helped encourage physical health changes and improved mental health. Our findings of mental and physical benefits support the previous qualitative insights from the Primrose trial [16] and wider research on the interrelated nature of mental health and CVD risk factors [31].

Due to the scarcity of integrated services, there is also limited research into factors that influence their implementation and delivery. The current research takes some steps towards addressing this research gap by providing insights into patient and staff experiences, including perceptions of delivery. Several barriers and facilitators were highlighted which could inform the future implementation of Primrose-A and similar complex interventions, which are discussed below.

Short appointment times and lack of continuous care are common barriers to SMI patients receiving appropriate physical healthcare [8]. Primrose-A addressed these barriers by providing longer regular appointments, which has previously been suggested to aid in building positive therapeutic relationships [32]. Primary care services operate with limited resources and time, preventing patients receiving mental health and cardiovascular care in one appointment [33]. The time and resource investment of Primrose-A was a common concern among staff when considering long-term implementation. Whilst the extended appointment time and regular contact provided through Primrose-A is a key strength, nurses highlighted that their time must be appropriately allocated to facilitate Primrose-A delivery, echoing qualitative findings from the Primrose intervention [16].

Moreover, in the current study, the strong patient-nurse/GP connection was suggested to encouraged patient engagement, trust, and motivation, which aligns with findings from the Primrose study [16] and adds to the evidence base that good patient-healthcare provider relationships can facilitate quality care [34]. Fostering a strong relationship can also be linked to addressing experiences of isolation for patients with SMI. It is well documented that patients with SMI experience isolation connected to their diagnosis and related factors such as unemployment, stigma, and lack of social participation [35]. Moreover, this study contributes to existing evidence [36, 37] that peer support may help patients to be more motivated to make positive health changes. Participants suggested relatability between patients and peer-coaches was key to the positive experience.

Developing therapeutic relationships was emphasised as important in the context of COVID-19. The COVID-19 pandemic had a detrimental effect on people’s mental health in the UK, leading to new psychoses and worsening of SMI symptoms [38]. Particularly recognised was the impact of social distancing measures on social isolation [39], which was commonly discussed in both patient and staff interviews. The current study found that providing Primrose-A appointments regularly supported in the perception of reduced isolation and anxiety, and in line with previous research found building social connection was beneficial [35].

Extensive literature documents how SMI diagnoses prevent access and engagement with healthcare [8], and in the Primrose study, low patient attendance of in-person appointments was reported [16]. This was not reported with Primrose-A, which may indicate that telephone appointments may better suit the needs of some patients. Whilst staff highlighted their preference for in-person delivery, telephone appointments were deemed acceptable by most patients, with some preferring this option when their SMI symptoms, such as experiences of anxiety and paranoia, had worsened. However, a limitation of telephone only consultations is the inability for staff to track physical health improvements in a clinically meaningful way. Despite this, these insights suggest the benefits of flexible intervention delivery. Internet-based platforms showed promising results in psychoeducation, improving initial engagement and treatment of mental health patients [40] possible explanation for this is that technology-based consulting avoids the patient ‘being seen’ seeking help, consequently reducing the associated embarrassment, social disapproval, and stigma [41]. For marginalised patients this may facilitate access to healthcare.

Sufficient time dedicated to adequate staff training was identified among Primrose-A staff, with evident disparities in staff experiences and skillsets. This aligns with findings from the original Primrose study, with the recommendation that staff be trained in both mental and physical health because of differences in their knowledge [16]. Staff education is also a widely employed tool to promote patient engagement in the population with SMI [42], and inadequate training and lack of confidence among primary care providers is a barrier to patients with SMI receiving appropriate medical care. Thus, more comprehensive staff training, including specialist training in substance abuse, would facilitate better patient care [30].

Additionally, staff described challenges with organisation and communication, with improvements to the planning and administrative aspects of Primrose-A suggested as required in future implementation. When considering the communication between staff and peer workers, this first needed staff delivering Primrose-A to engage with and embrace integrated working (both to incorporate mental and physical health care, and to integrate primary care staff and peer workers). Efforts to facilitate connections and a shared vision of Primrose-A across staff included shadowing and educational sessions, but embedding those with lived experience within the system was challenging and may be the foundation to the perceived poor communication and organisation. Therefore, further consideration of how to normalise peer worker integration rather than perceiving this role as a bolt on may be necessary to create a fully ‘boundary-spanning’ collaborative innovation [21].

Nevertheless, overall, staff were able to implement and deliver Primrose-A, and the flexibility and ability to build positive therapeutic relationships with Primrose-A facilitated patients’ positive experiences and goal achievement. Patients had a preference to extend the duration of Primrose-A, and staff to broaden the inclusion criteria to be able to deliver the intervention to more patients who may benefit, demonstrating the intervention’s acceptability.

### Implications for practice

These findings have implications for policymakers and the NHS, supporting the development of other physical health interventions for patients with SMI, as they highlight key considerations for their design and acceptability. Whilst the clinical efficacy of Primrose-A has not been evaluated in this study, qualitative insights are useful for evaluating complex interventions [43]. This type of integrated care was deliverable and accepted by patients and staff, and we suggest that our participants recognised the meaningful impacts of Primrose-A on mental and physical health.

This study indicated that the acceptability of Primrose-A was facilitated by strong therapeutic relationships with patients, regular provision of longer appointments, and flexible support in terms of goals, appointment content, and mode of delivery. Combining these insights with those from previous Primrose research [16, 18], continued implementation of Primrose-A across GP surgeries may offer improvements to quality of life for patients with SMI, as well as a cost-effective solution to the burden of SMI on NHS resources. But there is a need for further investigation into the clinical and cost-effectiveness of Primrose-A in reducing CVD risk, including comparison to the original Primrose intervention.

To improve the acceptability of Primrose-A and potentially support sustained health benefits, provision of more peer-coaching sessions and follow up Primrose-A appointments are recommended. Moreover, it is suggested that for future implementation of Primrose-A the set-up and administrative tasks are well organised; staff receive in-depth physical and mental health training with the addition of specialist training on substance addiction; clear communication is established between staff which could be potentially fostered through further integration of roles; and more staff are appointed to reduce the burden on nurse time and resources.

### Strengths and limitations

Following good practice [44], the use of patient and public involvement helped this study to be meaningful and relevant. However, despite this collaborative approach to research development, the resulting sample was small and consisted of volunteers who may have been more engaged or had more positive experiences with Primrose-A, and therefore may be unrepresentative of the target population. We speculate that the reason for low uptake may be due to the immense pressures that primary care staff have been under for some years meaning that staff could not take time to participate.

However, seeking saturation or generalisation is incompatible with reflexive thematic analysis, and therefore greater emphasis is placed on the strength of study design and achieving quality through data collection and analysis [45]. A good depth of data was achieved, and the research design allowed for the triangulation of perspectives across staff and patients. Moreover, this research was supported by a multidisciplinary team, including providing insight into theme generation which is suggested to increase the credibility of findings [46]. The researchers allocated sufficient time to engaged fully with the methodology and methods outlined in this paper, including thorough immersion in the data, and embracing reflexivity. The transparency of these processes in the dissemination of the research is supported through use of the consolidated criteria for reporting qualitative studies [47] (see additional file 3).

The COVID-19 pandemic added challenges to this research, with limitations of this study being the delay between Primrose-A delivery and when study interviews took place potentially introducing recall bias. The need to exclusively conduct telephone interviews rather than offering the option of face-to-face interviews, may have reduced accessibility of the research. However, for the latter point, telephone interviews were unlikely to affect the richness and quality of the data as there is evidence that that telephone interviews can be advantageous, perhaps through participants being more relaxed and honest through being in their home environment [48].

## Conclusion

The aim of this research was to explore with patients and staff the acceptability and feasibility of, and experiences with Primrose-A. Overall, this research demonstrates the acceptability of providing integrated physical and mental healthcare to patients with SMI. Successful implementation of Primrose-A and other similar interventions into primary care may depend on the development of strong therapeutic relationships with staff, sufficient mental and physical health training, providing adequate time and opportunities for patients to receive continuous care, and flexibility of intervention content and delivery. These elements require long term funding in order to maintain continuity of relationships to support the development of staff expertise and health gains to patients. These findings should inform further developments to Primrose-A as it is expanded across further localities in England, whilst contributing to research on the implementation of future integrated and complex primary care interventions in the NHS.

### Electronic supplementary material

Below is the link to the electronic supplementary material.

Supplementary Material 1: Additional File 1

Supplementary Material 2: Additional File 2

Supplementary Material 3: Additional File 3

## Data Availability

The datasets generated and/or analysed during the current study are not publicly available due to ethical approvals allowing only the research team to access the data, but the dataset is available from the corresponding author on reasonable request.

## Abbreviations

CVD: Cardiovascular disease
GP: General practitioner
NHS: National Health Service
SMI: Severe mental illness
