# Supplementary material for: An integrated primary care service to reduce cardiovascular disease risk in people with severe mental illness: Primrose-A - thematic analysis of its acceptability, feasibility, and implementation
Source: BMC Health Serv Res. 2024 Feb 28;24:255. doi: 10.1186/s12913-024-10628-6 (PMC10900648; doi:10.1186/s12913-024-10628-6)
Supplement: Supplementary file 1 — Supplementary Material 1: Additional File 1 [file 12913_2024_10628_MOESM1_ESM.docx]

# Additional File 1

## Patient interview topic guide

Introduction

- Thank you for taking part in this research, I appreciate you taking the time to talk to me.
- I am interested in your experiences of the PRIMROSE A intervention. There are no right or wrong answers, so please be honest and open about your views.
- I will use a digital audio recorder to accurately capture what you say. Everything you say is confidential. Any information from this interview that is used in writing up the study findings will be anonymised (your name will not be used at any point) so that there will be no way of anyone knowing who participated in the research.
- If you tell me something that makes me concerned for your safety, or someone else’s safety, then I would talk to you about what we could do to address this.
- Do you have any immediate thoughts or questions before we start?

Experience of using the intervention

a) How did you find out about the PRIMROSE intervention?

- How were the sessions arranged? (e.g. by phone/at an existing appointment)?
- Any difficulties?/anything that helped make accessing the intervention easier?
- Was this support something you felt you needed?

b) Can you tell me about the aim of the PRIMROSE appointments for you?

- Who explained things to you?/How was information given?/Could it have been explained better?

c) I’d like you to think about your appointment(s) with the nurse. What was a typical appointment with the nurse like for you?

- How did you find these appointments?
- What kinds of things did you do with the nurse? [Important to be clear they don’t have to give personal details about what they discussed]
- e.g. setting goals about diet, exercise, losing weight, advice on cutting down/stopping smoking, support services available, information on medication (statins/antihypertensives), reducing or stopping drinking alcohol etc.

d) Did you receive any appointments with a Peer-coach?

- If no, can you tell me why?
- If yes, what was a typical appointment with the peer-coach like for you?
- How did you find these appointments?
- What kinds of things did you do with the Peer-coach?

e) [if relevant] How did the nurse and peer-coach sessions compare?

- Were both helpful/necessary?
- What was the impact of having both on your experiences of the intervention?

f) Did the COVID-19 pandemic have any impact on your experience of the intervention?

- [If they used the intervention pre-COVID-19]
- How did you feel about any changes to the intervention (helpful/unhelpful in setting/meeting goals)?
- Could anything have been done differently to improve how the intervention was delivered during the pandemic?
- What was your experience of face-to-face, telephone/videoconference sessions? What are your thoughts about these different ways of meeting with the nurse or peer-coach?

g) Do you feel the intervention has had an impact on your health?

- Any impact on your physical health? Your mental health? Your general wellbeing?
- Any impact on your activities/day-to-day life? E.g. taking medication regularly?
- Any negative consequences or side effects?

h) Did you have all your appointments, or did you finish using the intervention early?

- What were your reasons for continuing / discontinuing [as relevant] with the intervention?
- If finished early, is there anything that might have helped you to continue with the sessions?

i) How satisfied have you been with the PRIMROSE intervention overall?

- Do you think that anything that could be improved?

j) Would you recommend this type of intervention to someone else?

- Can you tell me a bit more about why or why not?

k) How does the PRIMROSE intervention compare with the usual care you receive from your GP practice?

- How did it fit in with your usual care?
- Have you ever received any similar support?

l) Were you given the opportunity to involve anyone who supports you (e.g. a friend or family member) in the PRIMROSE intervention?

- If no, what are your thoughts on having a supportive person take part with you?
- If yes, how were they involved?
- Can you tell me about the impact of their involvement on your experience of the intervention?
- Could their involvement have been made any easier for them/you?

Conclude discussion

a) Is there anything that we have not talked about that you would like to raise?

b) Are there any other comments/questions or ideas?

- Thank participants
- Reimburse them for their time and travel expenses
- Ask if they have further questions about the study
- Explain next steps of the study

## Nurse/GP interview topic guide

Introduction

- Thank you for taking part in this research, on behalf of the evaluation team, I appreciate you taking the time to talk to me.
- I am interested in your experiences of setting up the PRIMROSE A intervention. There are no right or wrong answers, so please be honest and open about your views.
- I will use a digital audio recorder so that I can accurately capture what you say. Everything you say will be treated as confidential. Any information used from this interview in writing up our findings will be anonymized so that there will be little chance of being recognized from what is published.
- Do you have any immediate thoughts or questions before we start?

Intervention set up

a) What do you see as the purpose of the intervention?

- How does the intervention fit with the overall goals/activities of the Trust/GP practice?

b) How involved were you in setting up the intervention?

- What went well/less well?
- How could set up have been easier/more efficient/effective?

c) What are your thoughts on any factors that influenced the decision to implement PRIMROSE-A?

- What was your role in the decision and process?
- What barriers/facilitators were encountered?

d) What resources were needed to implement the intervention?

- What influenced your Trust’s/GP practice’s ability to implement the intervention?
- Staff capacity & capability?
- Cost and reimbursement structure?

e) What impact did the COVID-19 pandemic have on setting up/providing the intervention?

- how do you see things going forward?

Monitoring

a) What do you hope the benefits/advantages are of the intervention?

- For patients/the practice/practice staff/Trust?

b) What do you think might be the disadvantages?

- For patients/the practice/practice staff/Trust?

c) What kinds of impacts were you hoping the intervention would have?

- On patients, practices, wider services, Trust, staff?

d) What kind of information is needed to monitor the intervention?

- Has it been possible to collect this information?
- Why/why not?
- If not, what would help with this?

e) What are your thoughts on providing this intervention long term?

- How would it fit with usual care?
- Any systems/processes needed to enable long term provision?

f) What considerations would be needed to expand PRIMROSE-A to other practices or settings?

Conclude discussion

a) Is there anything that has not been discussed that you would like to raise?

b) Are there any other comments/questions or ideas?

- Thank participants
- Ask if they have further questions about the study
- Explain next steps of the study

## Peer-coach interview topic guide

Introduction

- Thank you for taking part in this research, on behalf of the evaluation team, I appreciate you taking the time to talk to me.
- I am interested in your experiences of delivering the PRIMROSE A intervention. There are no right or wrong answers, so please be honest and open about your views.
- I will use a digital audio recorder so that I can accurately capture what you say. Everything you say will be treated as confidential. Any information used from this interview in writing up our findings will be anonymized so that there will be little chance of being recognized from what is published.

Experience of delivering the intervention

a) How did you become involved in delivering PRIMROSE-A?

b) What do you see as the purpose of the intervention?

- Do you think it fulfilled this purpose?

c) How did you find the PRIMROSE A manual and the planned session structure?

- Were they easy to use? Anything that helped/prevented following the structure?
- Were there times when more flexibility was needed? If so, did you feel ready/willing/able to adapt the intervention to patient needs/contexts?

d) How accessible do you think the intervention is for service users?

- What helped/hindered people accessing the intervention?

e) What impact (if any) did the COVID-19 pandemic have on providing the intervention?

- How did you find phone/video calls compared to face-to-face meetings?

f) Can you describe a typical intervention appointment?

- Examples of what went well.
- Examples of what was more difficult.
- Was there anything you could/would have done differently?

g) How did you feel about delivering the intervention?

- Did it evoke an emotional response? If so, was this helpful or unhelpful?

Impact on service users

a) Can you give me an example of a service user who made progress, and what that was like?

- How/why do you think this occurred?
- Is there anything with hindsight might be done differently with this service user?

b) Can you give me an example of a service user who made little/no progress or became more unwell?

- How/why do you think this occurred?
- Is there anything with hindsight that might be done differently with this service user?

c) What factors do you think might have kept the service users motivated to attend appointments?

- How did you encourage service users to attend?
- Do you know of any factors about the intervention that prevented or discouraged attendance?

d) How did you find the work around setting recovery goals with patients (e.g. medication adherence, increase exercise, stop smoking)?

- What helped/hindered this type of work?

Training and skills

a) What did you think about the training?

- If helpful – how? If unhelpful - why not?
- Anything you would change/alter/improve?

b) Did the training provide the skills/information needed to deliver the intervention?

- Mental health/Physical health (smoking cessation, diet)/Medications.
- Do you feel you have acquired any new skills you could use in the future?

Intervention administration, planning and monitoring

a) What influenced your ability to provide the intervention?

- E.g. access to support from team, colleagues, GP, other PRIMROSE nurses?

b) What kind of information did you record about the intervention?

- How was this information recorded and monitored?
- Was there any additional information that you think should have been recorded?

c) How did you find supervision?

- Helpful/unhelpful?
- What helped/didn’t help?
- What could be improved about supervision?

Ending the Intervention

a) How did you feel about ending the intervention & finishing the sessions with service users?

- What did your service users feel about it coming to an end?
- What kinds of things were discussed at the final appointment?

Conclude discussion

a) Is there anything that has not been discussed that you would like to raise?

b) Are there any other comments/questions or ideas?

- Thank participants
- Ask if they have further questions about the study
- Explain next steps of the study
