# Supplementary figures and images for: An integrated primary care service to reduce cardiovascular disease risk in people with severe mental illness: Primrose-A - thematic analysis of its acceptability, feasibility, and implementation
Source: BMC Health Serv Res. 2024 Feb 28;24:255. doi: 10.1186/s12913-024-10628-6 (PMC10900648; doi:10.1186/s12913-024-10628-6)

# Additional File 2

##
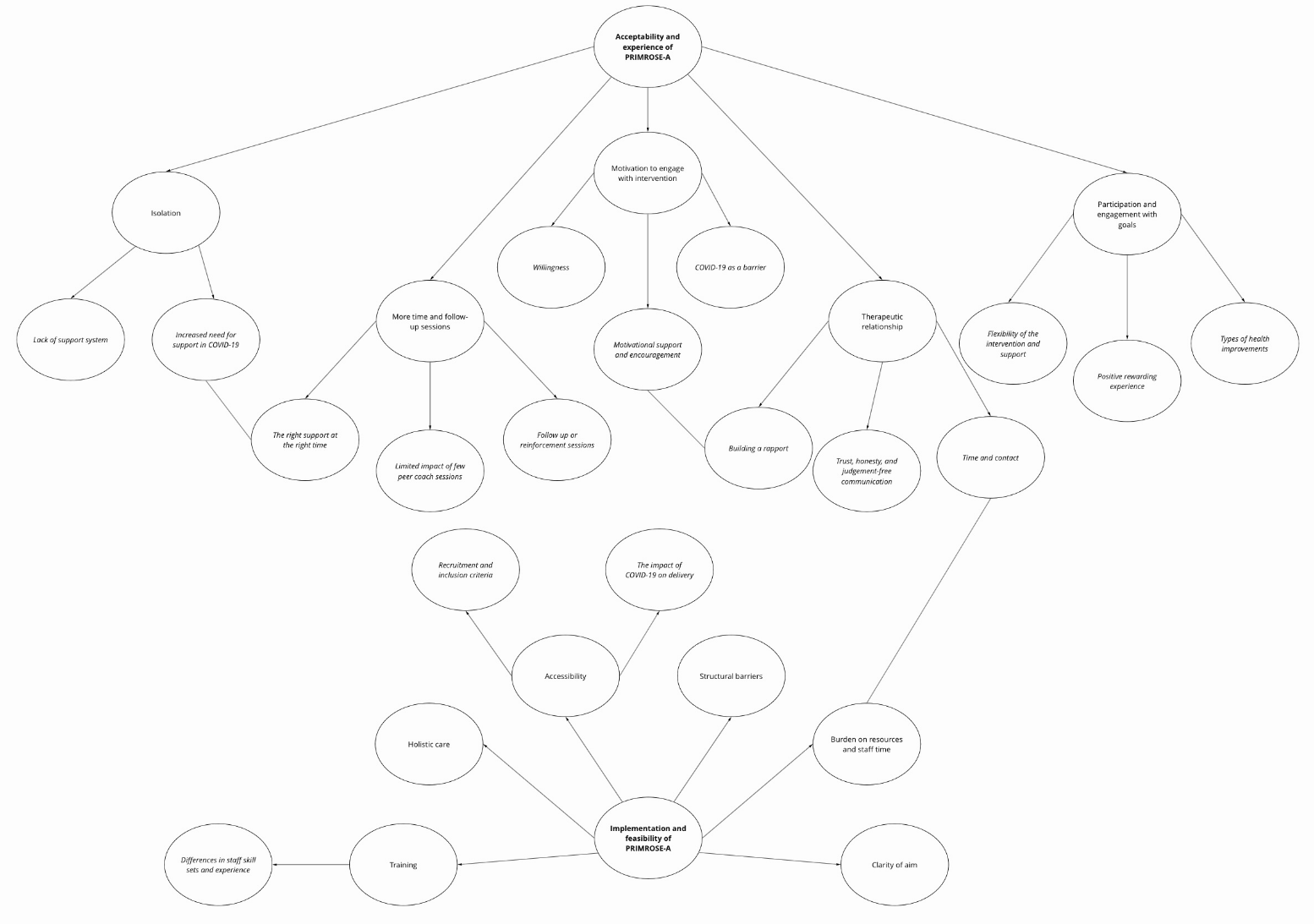
Thematic map of themes and subthemes

Supplement: Supplementary file 2 — Supplementary Material 2: Additional File 2 [file 12913_2024_10628_MOESM2_ESM.docx]
