# Supplementary material for: An integrated primary care service to reduce cardiovascular disease risk in people with severe mental illness: Primrose-A - thematic analysis of its acceptability, feasibility, and implementation
Source: BMC Health Serv Res. 2024 Feb 28;24:255. doi: 10.1186/s12913-024-10628-6 (PMC10900648; doi:10.1186/s12913-024-10628-6)
Supplement: Supplementary file 3 — Supplementary Material 3: Additional File 3 [file 12913_2024_10628_MOESM3_ESM.docx]

# Additional File 3

## Consolidated criteria for reporting qualitative studies (COREQ): 32-item checklist ^50^

| **Item** | **Guide questions/description** | **Reported on page** |
| --- | --- | --- |
| **Domain 1: Research team and reﬂexivity** | | |
| *Personal Characteristics* | | |
| 1. Interviewer | Which author/s conducted the interview or focus group? | p. 8, line 126 |
| 2. Credentials | What were the researcher’s credentials? E.g. PhD, MD | p. 8, line 126 |
| 3. Occupation | What was their occupation at the time of the study? | p. 8, line 126 |
| 4. Gender | Was the researcher male or female? | p. 9, lines 152-153 |
| 5. Experience and training | What experience or training did the researcher have? | NA – related to occupation (MSc student and supervisor) |
| *Relationship with participants* | | |
| 6. Relationship established | Was a relationship established prior to study commencement? | p. 8, line 115 |
| 7. Participant knowledge of the interviewer | What did the participants know about the researcher? e.g. personal goals, reasons for doing the research | p. 8, line 115 |
| 8. Interviewer characteristics | What characteristics were reported about the inter viewer/facilitator? e.g. Bias, assumptions, reasons and interests in the research topic | p. 9, lines 143-153 |
| **Domain 2: study design** | | |
| *Theoretical framework* | | |
| 9. Methodological orientation and Theory | What methodological orientation was stated to underpin the study? e.g. grounded theory, discourse analysis, ethnography, phenomenology, content analysis | p. 8, lines 129-134 |
| *Participant selection* | | |
| 10. Sampling | How were participants selected? e.g. purposive, convenience, consecutive, snowball | pp. 7-8, lines 110-116 |
| 11. Method of approach | How were participants approached? e.g. face-to-face, telephone, mail, email | pp. 7-8, lines 110-115 |
| 12. Sample size | How many participants were in the study? | p. 8, line 116 |
| 13. Non-participation | How many people refused to participate or dropped out? Reasons? | NA |
| *Setting* | | |
| 14. Setting of data collection | Where was the data collected? e.g. home, clinic, workplace | NA – telephone interview (p. 8, line 113) |
| 15. Presence of non-participants | Was anyone else present besides the participants and researchers? | NA |
| 16. Description of sample | What are the important characteristics of the sample? e.g. demographic data, date | p. 8, lines 116-121 |
| *Data collection* | | |
| 17. Interview guide | Were questions, prompts, guides provided by the authors? Was it pilot tested? | See additional file 1. |
| 18. Repeat interviews | Were repeat interviews carried out? If yes, how many? | NA |
| 19. Audio/visual recording | Did the research use audio or visual recording to collect the data? | p. 8, lines 124-125 |
| 20. Field notes | Were ﬁeld notes made during and/or after the interview or focus group? | NA |
| 21. Duration | What was the duration of the interviews or focus group? | p. 8, line 127 |
| 22. Data saturation | Was data saturation discussed? | p. 21, lines 400-410 |
| 23. Transcripts returned | Were transcripts returned to participants for comment and/or correction? | NA |
| **Domain 3: analysis and ﬁndings** | | |
| *Data analysis* | | |
| 24. Number of data coders | How many data coders coded the data? | p. 9, line 135 |
| 25. Description of the coding tree | Did authors provide a description of the coding tree? | Additional file 2 |
| 26. Derivation of themes | Were themes identiﬁed in advance or derived from the data? | p. 9, lines 135-138 |
| 27. Software | What software, if applicable, was used to manage the data? | p. 9, line 136 |
| 28. Participant checking | Did participants provide feedback on the ﬁndings? | p. 9, lines 137-139 |

| *Reporting* | | |
| --- | --- | --- |
| 29. Quotations presented | Were participant quotations presented to illustrate the themes/ﬁndings? Was each quotation identiﬁed? e.g. participant number | pp. 10-15 |
| 30. Data and ﬁndings consistent | Was there consistency between the data presented and the ﬁndings? | pp. 10-15 – integrated findings and commentary |
| 31. Clarity of major themes | Were major themes clearly presented in the ﬁndings? | p. 10, lines 158-161  p. 12, lines 212-216 |
| 32. Clarity of minor themes | Is there a description of diverse cases or discussion of minor themes? | pp. 10-15 |
